# Solid Organ Transplant and Parasitic Diseases: A Review of the Clinical Cases in the Last Two Decades

**DOI:** 10.3390/pathogens7030065

**Published:** 2018-07-31

**Authors:** Fabiani Silvia, Fortunato Simona, Bruschi Fabrizio

**Affiliations:** 1 Infectious Disease Department, Azienda Ospedaliera Pisana, 56124 Pisa, Italy; fabiani.silvia@libero.it; 2 School of Infectious Diseases, Università di Pisa, 56124 Pisa, Italy; simona.fortunato13@gmail.com; 3 Department of Translational Research, N.T.M.S., Università di Pisa, 56124 Pisa, Italy

**Keywords:** parasitosis, solid organ transplant (SOT), post-SOT parasitic infections

## Abstract

The aim of this study was to evaluate the occurrence of parasitic infections in solid organ transplant (SOT) recipients. We conducted a systematic review of literature records on post-transplant parasitic infections, published from 1996 to 2016 and available on PubMed database, focusing only on parasitic infections acquired after SOT. The methods and findings of the present review have been presented based on the Preferred Reporting Items for Systematic Reviews and Meta-Analysis (PRISMA) checklist. From data published in the literature, the real burden of parasitic infections among SOT recipients cannot really be estimated. Nevertheless, publications on the matter are on the increase, probably due to more than one reason: (i) the increasing number of patients transplanted and then treated with immunosuppressive agents; (ii) the “population shift” resulting from immigration and travels to endemic areas, and (iii) the increased attention directed to diagnosis/notification/publication of cases. Considering parasitic infections as emerging and potentially serious in their evolution, additional strategies for the prevention, careful screening and follow-up, with a high level of awareness, identification, and pre-emptive therapy are needed in transplant recipients.

## 1. Background

Solid organ transplantation (SOT) is a life-saving procedure, but it does not lack short-term and/or long-term risk. Despite continued improvement in the clinical management of SOT recipients, infections still represent one of the leading causes of morbidity and mortality in this population.

**Figure 1:**
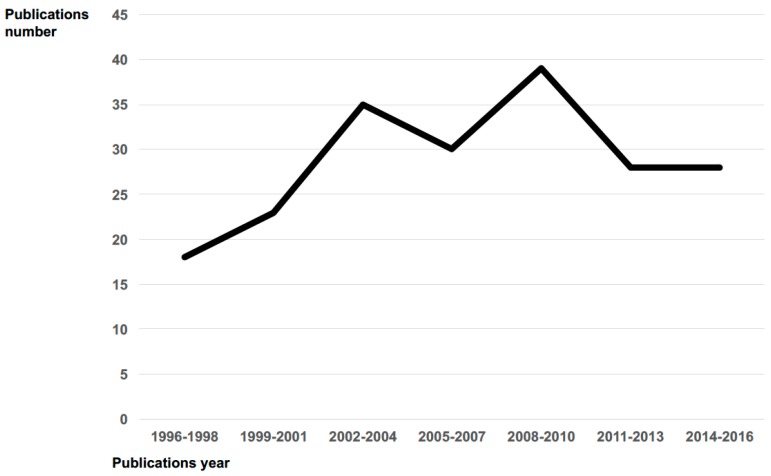
Publications number trend on parasitosis in SOT recipients per period 1996–2016.

SOT recipients can develop bacterial, fungal, viral, and/or parasitic infections. Although the prevalence of parasitic infections in SOT recipients was lower than viral, bacterial or fungal [1], in the last decade, there has been a growing identification of parasitic infectious diseases occurring in transplant hosts both in endemic and non-endemic settings for the specific parasitosis [2] (see Figure 1). This is probably due to more than one reason: (i) the increasing number of patients transplanted and then treated with immunosuppressive agents; (ii) the “population shift” resulting from immigration and travels to endemic areas, and (iii) the increased attention directed to diagnosis/notification/publication of cases.

Most of the data was published by European and North American Countries.

Parasitic diseases may affect transplant recipients as a result of (i) transmission by transplanted organ (or blood product, either before or after transplantation); (ii) reactivation or recrudescence of latent infections in the previously infected recipient; (iii) de novo infection in the post-transplant period by means of natural infection [2,3].

Reactivation of a dormant infection occurs as a consequence of immunosuppression which in transplanted subjects, is induced by drugs required to induce immunosuppression in early phase and to maintain immunosuppression in the late phase or to treat organ rejection. Different therapeutic strategies are nowadays available: corticosteroids, antibodies, calcineurin inhibitors (CNI), anti-metabolite agents and mammalian target of rapamycin inhibitors (mTORI), depending on the protocols used by the different transplant centers, distributed worldwide. The optimal level of immunosuppression in SOT is a delicate balance between the rejection’s prevention and the immunosuppression’s side effects. As a consequence, all SOT recipients need an individually tailored immunosuppression regimen [3].

## 2. Objectives

We conducted a systematic review of literature records on post-transplant parasitic infections, published since 1996 to 2016 and available on the PubMed database, to analyze the occurrence of parasitic infections in SOT recipients.

## 3. Materials and Methods

**Figure 2:**
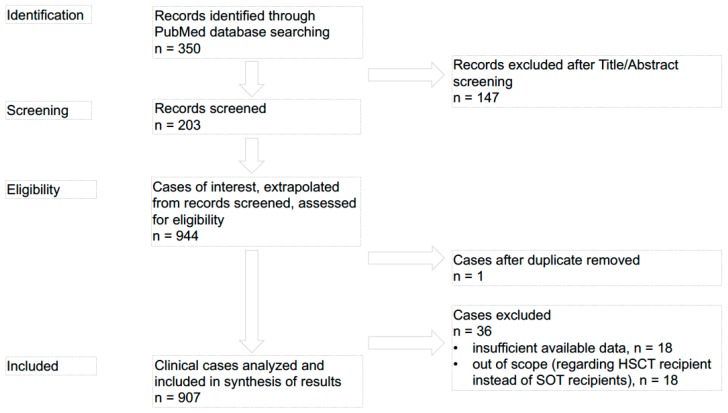
PRISMA flow chart: data collection and selection of studies.

The methods and findings of the present review have been reported based on the preferred reporting items for systematic reviews and meta-analysis checklist (PRISMA). Figure 2 provides a flow chart of the procedure used to collect information for the present review.

We searched the literature on PubMed library combining the term “solid organ transplant” or “SOT” along with the name of parasite genus or disease. The PubMed database was searched for papers published from 1 January 1996 to 31 December 2016.

No language restrictions have been used.

Two investigators (SF, SF) independently reviewed the retrieved articles in two stages; first assessing relevance from the title and abstract and if relevance was still unclear, the full text was read. Any disagreement about inclusion was referred to a third reviewer (FB) and resolved by discussion. From each eligible document, data were collected in predefined tables. The predefined tables summarizing individual cases included age, gender, Country of origin and/or nationality, and reported risk factors (e.g., immigration/travel history) for both donor and recipient of SOT, manner of parasitic acquisition, type of transplanted organ, type of immunosuppression, parasitic disease onset from transplantation, diagnosis of parasitic disease by clinical features, imaging and laboratory, parasitic disease treatment, outcome, and reference.

Papers were excluded if at least one of the following criteria were present: (i) records did not regard parasitosis in SOT recipients; (ii) transplant of known infected organ; (iii) studies published before 1996 or after 31 December 2016; (iv) studies reported insufficient data, required to complete predefined tables.

In cases where the existence of duplicates was probable, according to anamnestic, laboratory and clinical data, cases were only presented once. Descriptive analyses and graphics were performed in Excel.

## 4. Results

We identified 350 records overall. According to exclusion criteria i, ii and iii, 147 records were removed after Title/Abstract screening. Finally, we screened 203 records. From these records we extrapolated 944 cases of interest assessed for eligibility. One case, after duplicate, was removed, and 36 cases were excluded according to exclusion criteria i (out of scope) and iv (insufficient available data). In the present review we only analyzed cases regarding SOT recipients published from 1996 to 2016 and available on the PubMed database with sufficient data for inclusion on synthesis of results (n 907) [4,5,6,7,8,9,10,11,12,13,14,15,16,17,18,19,20,21,22,23,24,25,26,27,28,29,30,31,32,33,34,35,36,37,38,39,40,41,42,43,44,45,46,47,48,49,50,51,52,53,54,55,56,57,58,59,60,61,62,63,64,65,66,67,68,69,70,71,72,73,74,75,76,77,78,79,80,81,82,83,84,85,86,87,88,89,90,91,92,93,94,95,96,97,98,99,100,101,102,103,104,105,106,107,108,109,110,111,112,113,114,115,116,117,118,119,120,121,122,123,124,125,126,127,128,129,130,131,132,133,134,135,136,137,138,139,140,141,142,143,144,145,146,147,148,149,150,151,152,153,154,155,156,157,158,159,160,161,162,163,164,165,166,167,168,169,170,171,172,173,174,175,176,177,178,179,180,181,182,183,184,185,186,187,188,189,190,191,192,193,194,195,196,197,198,199,200,201,202,203]. Previously, allogeneic HSCT and parasitic infections were reviewed and discussed [204].

**Figure 3:**
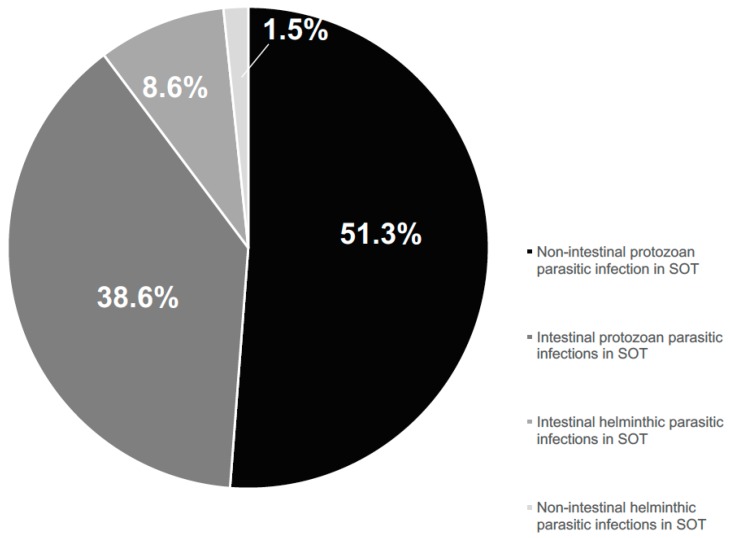
Parasitosis reported cases among SOT recipients by infectious agent (protozoa or helminths) (PubMed published data per period 1996–2016).

We summarized all literature reports on parasitosis after SOT on the basis of the parasite involved (non-intestinal protozoa, intestinal protozoa, intestinal helminths, and non-intestinal helminths) (Figure 3), grouping by: (i) type of transplanted organ (Table 1); (ii) country of origin or nationality, and reported risk factors (e.g., immigration/travel history) for both donor and recipient of SOT (Table 2); (iii) manner of parasitic acquisition (donor-derived through graft or transfusions, and primary infection by means of natural infection) and reactivation (Table 3); (iv) parasitic disease onset from transplantation (early 0–29 days, middle 30–100 days, and late >100 days) (Table 4); (v) laboratory diagnostic methods (direct and indirect methods performed in the ante- and/or post-mortem period) (Table 5); (vi) parasitic disease treatment (Table 6); (vii) patient outcome (Table 6).

## 5. Discussion

According to published literature and available data, parasitic infections represent a possible complication after SOT. Studies on this topic are evolving fields that are receiving increased recognition.

Transplant recipients may develop parasitic disease after acquisition of the parasite at the time of transplantation, either with the allograft or with blood and blood products, as well as in the post-transplantation period, through the traditional route of infection, or, as a consequence of immunosuppression, due to the reactivation of a dormant infection [2]. Indeed, many difficulties can often take place in the identification of the manner of acquisition of parasitic infections.

When parasitic infections occur in immunosuppressed people, they can manifest with several features. Clinical severity and outcome certainly depend on parasite features, innate and acquired host immunity as well as on immune interaction between parasite and host.

Blood parasites (e.g., *Plasmodium* spp., *Babesia*, *Schistosoma* spp.), tissue parasites (e.g., *Leishmania* spp, *Toxoplasma gondii*, *Trypanosoma cruzi*, *Acanthamoeba*), and intestinal parasites (e.g., *Microsporidia* spp., *Cryptosporidium* spp., *Entamoeba* spp., *Giardia duodenalis*, *Strongyloides stercoralis*, *Taenia solium*, *Trichuris trichiura*, *Ascaris lumbricoides*) can induce both localized syndrome and systemic illness. Due to the immunocompromised status of the host, the localized syndromes evolve into acute systemic illness more often than in the general population.

Prolonged fever alone or in combination with other systemic manifestations, anemia, lower gastrointestinal symptoms, and variable stigmata of organ involvement represent the most frequent clinical pattern, occurring in many parasitosis. Actually, it is difficult to identify and interpret all potential clinical patterns in which non-infectious events such as conditioning regimens, drugs, and acute GVHD may interfere.

Means of acquisition, clinical features, diagnostic methods and treatment measures of the most serious parasitic-specific infections reported in SOT recipients are listed below.

### 5.1. Non-Intestinal Protozoan Infections

**Toxoplasmosis** is a life-threatening opportunistic infection that may affect transplanted people. It is caused by the coccidian *Toxoplasma gondii*. Its occurrence in these patients is closely related to the prevalence of toxoplasmosis in the general population, which is high in Europe, but declining in recent decades [205,206].

In published and available reviewed literature, we found 162 case reports of toxoplasmosis occurring after SOT [4,5,6,7,8,9,10,11,12,13,14,15,16,17,18,19,20,21,22,23,24,25,26,27]. Of interest, in SOT recipients, toxoplasmosis results more frequently from transmission of the parasite with the transplanted organ from a *Toxoplasma*-seropositive donor (D+) to a *Toxoplasma*-seronegative recipient (R−). This risk is greater for transplantation of organs with high numbers of tissue cysts, e.g., the heart (see muscles sustaining parasite encystment), and it is markedly lower for the other organs [207]. Transmission of *T. gondii* from a D+ to an R+ may also occur. In this case, graft transmission is difficult to confirm and to differentiate from a reactivation of latent infection in the recipient. However, the hypothesis of reinfection of an R+ from a D+ has been suggested by Robert-Gangneux et al. [207]. In this study, western blot (WB) analysis of post-transplant sera of R+ showed neosynthesized IgG, probably related to the recognition of the new parasite strain acquired via the transplanted organ from a D+. This reinfection could be proved only with the identification of the infecting strain(s), by serotyping or genotyping [208,209,210].

In case of a recently infected donor, the possible presence of *T. gondii* in the blood represents a potential risk of transmission to an R−.

According to the literature data on post-transplant toxoplasmosis, transmission occurred through graft in 31.5% (n 51), de novo infection in 9.9% (n 16) and reactivation in 8% (n 13). However, in 50.6% (n 82) the modality of infection remained unknown.

As already said, although heart transplant is riskier for organ-related toxoplasmosis than liver, lung, or kidney transplant, data from the last decade published records showed kidney transplant as the most frequently implicated in post-transplant toxoplasmosis (n 75, 46.3%), followed by heart (n 55, 34%). Liver (n 19, 11.7%), bowel, pancreas, lung and simultaneous multivisceral (few cases) transplants have also been reported.

Toxoplasmosis in the immunocompromised host presents with pyrexia, lymphadenopathy, and multiorgan involvement. Anemia is common, and a hemophagocytic syndrome has been reported in several cases [211]. Encephalitis, meningoencephalitis, and cerebral mass lesions are serious and frequent complications [9,212]. Chorioretinitis, similar to that observed in Cytomegalovirus (CMV) infection, frequently occurs [25,213]. Myocarditis and pneumonitis are also reported [7,13,214].

According to collected data, primary toxoplasmosis acquired through the graft is usually more severe than reactivation disease (mortality of 31.4% for graft-related toxoplasmosis versus 7.7% for reactivation cases) and in the case of graft transmission, it occurs much earlier than reactivation, often within 100 days of transplantation.

The serological status for infection with *T. gondii* in both the donor and recipient must be determined prior to transplantation. After transplantation, the diagnostic work ideally includes not only traditional serological assays but also sensitive techniques like comparative WB between pre- and post-transplant pattern [207] and polymerase chain reaction (PCR) in biological samples [215]. The study of cellular immune response using toxiferon, useful for the diagnosis of congenital toxoplasmosis [216], is under evaluation in transplanted individuals in few Centres, but to our knowledge no report is present in the literature about the use of such a method in the follow up of transplanted individuals. Moreover, the definitive diagnosis often requires the direct demonstration of parasites (e.g., histologically) or parasitic DNA in blood, bone marrow, cerebrospinal fluid (CSF), bronchoalveolar lavage (BAL) fluid or biopsy specimens. Due to the immunocompromised status of the patients, the serological tests could become less useful for the diagnosis, because of their reduced sensitivity in these conditions.

There is good evidence that in R− of organs from a D+, prophylaxis with trimethoprim-sulfamethoxazole (TMP-SMZ) immediately after transplant reduces the incidence of primary infection: particularly in heart transplant, the occurrence decreases from more than 50% without prophylaxis [217] to about 5% with prophylaxis [218]. However, fatal disseminated toxoplasmosis has been reported even in the case of seropositivity match, suggesting that prophylaxis should be extended also in these cases [13].

Seronegative candidates must be re-checked immediately before transplant in order to avoid unnecessary chemoprophylaxis, in case of seroconversion. All R− of organs from a D− should follow behavior and dietetic rules to avoid exogenous infection, and should be tested for *T. gondii* antibodies every six months. R− of organs from a D+ are treated with TMP-SMZ immediately after transplant; moreover, they should follow hygiene and dietetic rules such as those for recipients of organs from a D−.

In these patients, serological tests should be carried out at the end of chemoprophylaxis and then every six months. All patients, including those seropositive before transplant, should be tested for *T. gondii* serology when presenting with suggestive symptoms. Mild presentations and nonspecific symptoms should raise a suspicion of toxoplasmic disease, particularly after antireject treatment [14]. In cases of strong clinical suspicion, with or without laboratory diagnostic confirmation, full regimen treatment (TMP-SMZ or pyrimethamine-sulphadiazine, as standard treatmet, or clindamycin, clarithromycin, azithromycin or atovaquone, as alternatives) should be recommended [3].

Regarding treatment, some limitations exist because of potential drug to drug interaction between anti-*Toxoplasma* and immunosuppressive treatments. For example, sulfadiazine or SMX together with cyclosporine (CsA) or tacrolimus (TAC) increases risk of kidney damage, azithromycin together with TAC increases the risk of arrhythmia; sulfadiazine and clindamycin therapy can decrease CsA concentration [3]. On the other hand, the possibility that CsA possesses anti-*Toxoplasma* activity must be considered [3].

**Leishmaniasis** is a vector-borne tissue parasitic disease, localized (cutaneous and muco-cutaneous) or systemic (visceral leishmaniasis = VL), caused by the kinetoplastidae *Leishmania* genus. Cases are recorded among patients undergoing kidney, liver, heart, lung and pancreas transplantation, with the most significant association with kidney transplantation (84.1%, based on the 127 cases reported in the literature) [28,29,30,31,32,33,34,35,36,37,38,39,40,41,42,43,44,45,46,47,48,49,50,51,52,53,54,55,56,57,58,59,60]. VL is the most frequently observed clinical presentation, followed by mucosal and more rarely, cutaneous. Like other parasites, *Leishmania* spp. can cause asymptomatic infection and then remain dormant in the host for many years, becoming clinically apparent during periods of immunosuppression [219]. As a consequence, leishmaniasis must always be considered in the differential diagnosis of febrile immunosuppressed transplant recipients (especially if renal) with fever, pancytopenia with or without allograft dysfunction [219], especially in endemic areas. The diagnosis in immunosuppressed patients is difficult because of the variability of symptoms and clinical signs, and the poor sensitivity of serology in this setting. Aspiration and biopsy of the bone marrow is the preferred method to confirm infection in such individuals [219]. For kidney transplantation, renal biopsy is also required in the case of graft dysfunction, allowing differential diagnosis between graft rejection, drug-related nephrotoxic lesions and parasitic parenchyma infiltration. The pre-operative check-up with serological testing for leishmaniasis of both transplant patients and donors and the regular post-transplant serological monitoring (although with limitations due to immunosuppressive status) of recipients should be performed to identify people at higher risk of leishmaniasis. Primary prophylaxis is not routinely used, but infection outcome depends on early diagnosis and effective antiparasitic therapy [55]. Treatment choice is conditioned by toxicity and drug interactions, which is even more important in these patients [55]. For this reason, liposomal amphotericin B may be considered the treatment of choice of VL, in consideration of the low incidence of side effects [3].

### 5.2. Chagas’ Disease

Chagras’ disease is another vector-borne parasitic disease, but caused by the American *Trypanosoma*, which has a very important impact on public health in Latin America [220].

Among the different ways of transmission, that with transplanted organs is reported along with reactivation of dormant infection in transplant recipients. In particular, of 88 SOT recipients described in literature as having trypanosomiasis, 29 (32.9%) had a demonstrated primary infection due to well established transmission through allograft; 49 (55.7%) had a reactivation, 2 (2.3%) had de novo infection, and 8 (9.1%) did not have a determined mechanism [15,61,62,63,64,65,66,67,68,69,70,71,72,73,74,75,76,77,78,79,80,81,82]).

The most significant association is reported with heart transplantation (60.2%, based on the 53 cases reported in the literature), with kidney transplantation second (22.7%, based on the 20 cases reported in the literature).

Indeed, *Trypanosoma cruzi* itself is responsible for a significant proportion of end-stage cardiomyopathy and heart transplant for Chagas’ heart disease should be regarded as a valuable treatment option [221]. Reactivation in Chagasic heart transplant recipients has been reported to occur in between 26.5% [66] and 42.9% [221] of patients with great variability among transplant Centers.

In reported data in this review, *Trypanosoma cruzi* disease after heart transplantation occurred for reactivation in more than 80% (n 43/53) of cases.

Early diagnosis is needed [73,222], because of the high rate of morbidity and possible mortality, which are not however more relevant than in non-infected transplanted subjects. Indeed, survival probability for Chagasic heart transplant recipients at 1 month, 1 year, 4 year, and 10 year follow-ups are 83%, 71%, 57%, and 46%, respectively [65,221], better than that seen in non Chagas’ heart transplant recipients [221].

Like the native disease, acute, chronic, and reactivated trypanosomiasis in transplant recipients manifests with variable pattern from asymptomatic forms to life-threatening problems. Fever, myalgia, lymphadenopathy, hepatosplenomegaly and subcutaneous nodules are the most prevalent manifestations; less commonly meningoencephalitis and myocarditis can occur during the acute phase, while potentially lethal cardiomyopathy, megasyndrome (megaesophagus/megacolon), or both, can occur during the chronic phase. Complete heart block is a possibility, presenting clinical feature in patients with reactivation.

Diagnosis involves detection of circulating parasites by microscopic examination and blood culture in the acute phase, and by serology thereafter [73]. For heart transplantation, endomyocardial biopsy allows differentiation between graft rejection and parasitic disease. The pre-operative check-up with serological testing for *T. cruzi* of both transplant patients and donors is standard practice: [73] even asymptomatic persons testing positive are probably infectious for life, with low levels of parasite in blood and other tissues [73]. In case of mismatch donor/recipient, follow-up schedule and pre-emptive therapy must be performed to abate the parasitaemia and avoid clinical illness [65].

Benznidazole is the treatment of choice [3]. Nifurtimox is an effective alternative [3].

**Malaria***Plasmodium* life cycle with a hepatic and an erythrocyte stage, allows the transmission of infection either with SOT or with blood transfusion, respectively. In such a setting, malaria remains a rare complication. However, post-transplantation malaria, although uncommon, must be considered particularly when either the recipient or the donor comes from a region endemic to malaria [90].

Primary infection or reinfection is a distinct risk in exposed transplant recipients. In published and available literature, post-transplant malaria reported cases involve kidney (16 cases, 59.3%), liver (5 cases, 18.5%), and heart (2 cases, 7.4%) [83,84,85,86,87,88,89,90,91,92]. Among these patients, 13 had a primary infection due to well clarified transmission through allograft (n 11, 40.7%) or blood/blood products (n 1, 3.7%), and 1 patient (3.7%) had a documented de novo infection. Nevertheless, in most of them the mechanisms of transmission were not clearly determined (n 14, 51.8%).

The clinical picture of malaria in transplant recipients is usually severe, owing to the impaired immune response. It is characterized by pyrexia, which may lack the typical periodicity or rigors. Anemia is severe, being typically hemolytic and occasionally hemophagocytic. It is often associated with thrombocytopenia [86]. Hepatosplenic γδ-cell lymphoma probably attributed to malarial infection, has been described in kidney transplant recipients [223]. Acute graft dysfunction may occur as a result of the hemodynamic consequences of *P. falciparum* infection [224]. Whether the immune response to malarial infection has an impact on subsequent rejection is unknown. Diagnosis is confirmed by examination of a Giemsa- or acridine orange-stained peripheral blood smear. When parasitaemia is low, serological techniques using synthetic peptides as antigen [225] or DNA probes [226] are useful for diagnosis.

Antimalarial drugs can be used safely in most patients without incurring problems. However, certain drug-drug interactions must be taken into consideration such as those between quinine and chloroquine with CsA: quinine decreases CsA blood levels, chloroquine increases CsA blood levels [3]. This may be extrapolated to other immunosuppressive agents which depend on cytochrome P450 for their catabolism. Moreover, TAC together with chloroquine, artemisin combinations, or mefloquine increases the risk of arrhythmia [3].

Providing routine malaria prophylaxis probably is not necessary for SOT recipients on maintenance immunosuppressives [227].

In addition, a certain protective anti-malarial activity can be postulated from in vitro results obtained with immunosuppressive drugs, such as sirolimus and CsA. Instead, for the moment no conclusive data are available for everolimus and mycophenolic acid if tested at standard doses for the clinical use [3,228].

Beyond any possibility of tailored treatment (specific anti-malarial and selected immunosuppressive combined treatment), prevention and early individuation of at risk patients remains the most important measure.

In this context, careful anamnesis on epidemiological risk has to be seriously considered for each donor and recipient in both malaria endemic and non-endemic zones, considering that infection can still be acquired in non-endemic locations including European or American airports or autochthonous malarial foci [229,230].

**Babesiosis** is a rare febrile disease, closely related to *P. falciparum* malaria, caused by piroplasmia *Babesia*, transmitted usually by tick bites. Babesiosis, attributed to graft or transfusion with contaminated blood, has been reported in four SOT recipients, three renal [94,96] and one cardiac [95]. In a further renal recipient affected by babesiosis, the mechanism of transmission has not been well clarified [93]. Fever, hemolytic anemia, and impaired graft function dominate the clinical picture in the kidney transplanted patient who acquired babesiosis through blood transfusion [94]; fever, fatigue and abdominal pain have been found in the two kidney transplanted patients acquiring parasitosis through the graft [96]; acute respiratory distress in the heart transplanted patient [95]; hemophagocytic syndrome has been reported in the latter, an asplenic renal transplant recipient [93]. Treatment consists of a combination of clindamycin and quinine, with therapeutic apheresis in severe cases [3].

**Acanthamoebiasis** is a protozoal disease caused by free-living amoebae.

*Acanthamoeba castellanii* typically complicates corneal transplantation leading to progressive keratitis, corneal opacities, or perforation [97]. It has also been reported in kidney [98,99,108], liver [105], lung [101,107], and multivisceral transplantation [100]. The total number of reported cases of post-SOT *Acanthamoeba* spp. infection amount to 17 cases. Disseminated acanthamoebiasis in transplant recipients is associated with gastroenteritis, sclerosing cholangitis, encephalitis [100,101,107], and osteomyelitis [99]. A fatal outcome has been reported in most disseminated cases.

Free-living ameba *Balamutia mandrillaris* has been reported as a cause of granulomatous amebic encephalitis. Infection with transplant-transmitted *B. mandrillaris amebae* was first identified by CDC reporting two clusters of infections in 2009 [103] and 2010 [104].

*Balamuthia* is known to spread hematogenously from extra-central nervous system (CNS) sites to the CNS [225]. Moreover, the described clusters of transplant transmission confirm that hematogenous spread of *Balamuthia* occurs from the CNS to other organs as well [231].

*Lophomonas blattarum* potentially leads to post-transplantation broncopneumonia. Initial symptoms can be relatively obscure. Thus, possible *L. blattarum* infection needs to be screened in patients with respiratory symptoms, especially in those who respond poorly to anti-infection treatment [102,106].

**Intestinal protozoan parasitosis** seems to be more common among transplant recipients compared to non-transplanted control subjects [134]: even parasites that are largely asymptomatic before transplantation may become clinically evident under immunosuppressive treatment.

Gastrointestinal disturbances, particularly diarrhea, both acute and chronic, are frequently observed complications in the first months following transplantation, potentially leading to a deterioration of the general health status in transplanted people. It is difficult to determine the exact etiology of the diarrhea in these patients, including drug-specific effects (i.e., mycophenolate mofetil, antibiotics, colchicines, laxative drug), metabolic conditions, mechanical complications of surgery, acute GVHD, as well as infectious agents (bacteria, such as *Clostridium difficile*, *Campylobacter jejuni*, *Shigella sonnei* and *Salmonella enteritidis* and virus, first of all CMV, but also Rotavirus, and parasites, in particular protozoan agents such as *Cryptosporidium* spp., *Blastocystis* spp., *Giardia duodenalis*, *Entamoeba histolytica*, *Entamoeba coli*, *Endolimax nana*, *Iodamoeba butschili*, *Chilomastix mesnili*, and *Microsporidia* spp.) [110,111,112,113,114,115,116,117,118,119,120,121,122,123,124,125,126,127,128,129,130,131,132,133,134,135,136,137,138,139,140,141,142,143,144,145,146,147,148,149,150,151,152,153,154,155,156,157,158,159,160].

In this scenario, the screening of samples for intestinal parasitic infections using direct smear, formalin-ether sedimentation, Sheather’s flotation and modified Ziehl-Neelsen staining methods acquires extreme importance [232].

Colonoscopy with biopsy should be performed only after the implementation of available noninvasive testing for infectious diarrhea and upon evaluation of all medications taken, to assess the possible cause of diarrhea [114,131,232,233].

Intestinal protozoa can be difficult to eradicate even with specific treatment. Reduction in immunosuppressive regimen may hasten clearance of these durable pathogens [3].

Beyond localized syndrome, peri-transplant acquired or reactivated enteric protozoan parasitosis can also manifest as extra-intestinal (e.g., hepatobiliary) and systemic diseases because of their aggravation and/or dissemination [232].

**Helminthic intestinal infections** should be actively evaluated in solid organ transplant recipients in both pre- and post-transplant phase.

On the basis of published data, the most frequently intercurrent helminthic intestinal infection in SOT recipients seems to be strongyloidiasis [15,161,162,163,164,165,166,167,168,169,170,171,172,173,174,175,176,177,178,179,180,181,182,183,184,185,186,187,188,189,190,191], being occasionally reported infections by other intestinal helminths such as *Trichuris trichiura* [192], *Ascaris lumbricoides* [134,193] and *Dipylidium caninum* [194].

As **strongyloidiasis** is a devastating complication of immunosuppression, SOT recipients can experience serious disease up to death from infection due to such a parasite.

Transplant recipients are at highest risk during the first three months post-transplant. Many organ systems may be affected, particularly in the case of autoinfection evolving in disseminated infection often complicated by polymicrobial sepsis due to enteric organisms adhering to the parasite.

Mortality is placed over 30% [15,161,162,163,164,165,166,167,168,169,170,171,172,173,174,175,176,177,178,179,180,181,182,183,184,185,186,187,188,189,190,191], thus careful evaluation of patients at any risk of exposure is essential. Stool specimens as well sputum, urine, and duodenal aspirates may be examined for characteristic organisms. It has been suggested that stool examination become part of the pre-transplantation work-up in all patients, with a more extensive evaluation in patients with a history of travel to, or residence in, an area of endemic infection.

Dormant infection for over 10–20 years must also be considered. Prolonged corticosteroid use, either in the early post-transplant period or during treatment of rejection episodes, may reactivate dormant strongyloidiasis and promote its diffusion in infected hosts.

Regarding the treatment, ivermectin has been increasingly used, being effective, and even more so, tolerated, it represents the first line treatment [3]. Repeat courses may be needed to eradicate infection [3]. Albendazole represents the alternative treatment [3]. The use of CsA in prophylactic immunosuppression seems to reduce strongyloidiaisis occurrence thanks to a strong parasiticidal effect of the drug, which has been documented in mice and humans [3]. Conversely, tacrolimus seems to increase the risk of infection [3]. Consequently, treatment decisions should be made on a case-by-case basis.

***Taenia solium*** larva is the most common parasite affecting the CNS. It is unusual in transplant recipients, but the risk must be taken into account: neurocysticercosis (NCC) must be included in the differential diagnosis of patients with CNS involvement and intracranial space-occupying lesions (SOLs) along with tuberculosis (TB), toxoplasmosis, nocardiosis, fungal infections, and post-transplant lymphoproliferative disorder (PTLD) [23,234]. The temporal association of the infection with the time elapsed since transplantation, risk factors, clinical manifestations, and neuroimaging characteristics of the lesion can allow a reasoned and rational approach towards the recognition, diagnosis, and appropriate management [195,196,234].

### 5.3. Non-Intestinal Helminths

Schistosomes inhabit the portal (***S. mansoni***) or perivesical (***S. haematobium***) veins of humans often living silently in an infected host for decades. During schistosomular migration through the lymphatics to the bloodstream and/or the permanence of adult worms in the gastrointestinal and urinary tracts, the disease may be theoretically transmitted by blood transfusion or organ transplantation. Considering that patients with schistosomal infection are suitable recipients for kidney and liver transplantation, recrudescence is also possible. On the other hand, transplant recipients may be exposed to new infection or reinfection if exposure to contaminated water persists.

Schistosomiasis is a well-recognized cause of chronic liver disease and non-cirrhotic portal hypertension. Ova of *S. mansoni* and *S. japonicum* trapped in the hepatic microcirculation induce inflammation and fibrosis (e.g., granulomatous hepatitis and portal or “clay pipe-stem” fibrosis) through both direct hepatotoxicity and immune mediated damage [197,198,235].

Schistosomiasis can also induce glomerular lesions through immune-complex deposits containing schistosomal gut antigens. As a consequence, patients with schistosomal infection are subject to the risk of urological complications [235].

However, schistosomal infection is not a major risk factor for transplantation [235]. Therefore, infected patients can be considered as suitable recipients. Nevertheless, careful selection of kidney donors and recipients with appropriate antischistosomal treatment for at least one month before transplantation are highly recommended [236,237].

As regards **echinococcosis (cystic and alveolar)**, transplantation of the involved organ should be regarded as a valuable treatment option. Recurrence inside and/or outside the transplanted organ is possible [199,200,201,202,203,237].

### 5.4. Perspectives: When Parasitic Infections Become Protective for Transplant Recipients

Several helminth-derived molecules have been able to modulate the host immune response [238].

Data obtained in animal model suggest that infection with the parasitic nematode *Trichinella spiralis* resulted in prolonged allograft survival following murine cardiac transplantation, with suppressed Th1/Th17 responses and augmented regulatory T cells [239].

Human data related to results of skin grafting in established schistosomiasis and more recent anecdotal suggestions of reduced immunosuppression requirement after liver transplantation for *Echinococcus* infection support the idea that helminth infection can enhance allograft tolerance. This is also demonstrated by multiple organ allograft animal models (e.g., mouse heart and skin; rat heart, liver, and kidney) [240]. As a consequence of such observations, the inclusion of specific live (non-pathogenic) helminth infection, or defined products from immunoregulatory helminths, in future transplantation protocols is now seriously considered as a potentially safe and effective alternative to current transplant immunosuppression regimens, burdened by multiple serious adverse effects and inadequate long-term organ protection against rejection [240]. Although treatment with active helminth infection has been shown to be a safe therapeutic approach [240], reports of mild gastrointestinal side effects do exist and might limit patient acceptability [241]. Further studies on this field are needed.

## 6. Conclusions

Parasitic infectious diseases after SOT, especially renal, emerge as possible and increasing complications, associated with significant morbidity and mortality. New guidelines should be elaborated focusing on the following: (i) diagnosis of active and latent parasitic infections, and identification of risk factors for candidate; (ii) recommended approach for parasitic infections diagnosed during the evaluation process and their corresponding treatment; (iii) definition of parasitic infections contraindicating transplantation; and (iv) prevention of post-transplantation parasitic infectious complications by systematic prophylaxis or pre-emptive treatment and instructions on preventive measures provided to patients, their relatives, and persons living with them. Using a multidisciplinary approach that included the efforts of experts in the field and the collaboration of scientific Societies, a comprehensive document containing specific recommendations should be elaborated.

## Figures and Tables

**Table 1:** Post-SOT parasitosis grouped by transplanted organ.

Infectious Agent	Post-SOT Parasitosis (Total Number of Reported Cases)	Post-SOT Parasitosis by Transplantation Type, n (%)									
		Kidney	Liver	Heart	Lung	Pancreas	Bowel	Iliac Vassel	Cornea	Multi-organ	Not Specified
**Non-intestinal protozoan parasitic infection in SOT (n 465)**											
***Toxoplasma gondii***	**162**	75 (46.3%)	19 (11.7%)	55 (34%)	1 (0.6%)	1 (0.6%)	1 (0.6%)			6 (3.7%)	4 (2.5%)
***Leishmania* spp.**	**151**	127 (84.1%)	13 (8.6%)	8 (5.3%)	3 (2%)						
***Trypanosoma cruzi***	**88**	20 (22.7%)	11 (12.5%)	53 (60.2%)	2 (2.3%)					2 (2.3%)	
***Plasmodium* spp.**	**27**	16 (59.3%)	5 (18.5%)	2 (7.4%)							4 (14.8%)
***Babesia***	**5**	4 (80%)		1 (20%)							
***Acanthamoeba* spp.**	**17**	13 (76.4%)	1 (5.9%)		1 (5.9%)		1 (5.9%)			1 (5.9%)	1 (5.9%)
***Balamuthia mandrillaris***	**9**	4 (44.5%)	2 (22.2%)	1 (11.1%)				1 (11.1%)		1 (11.1%)	
***Lophomonas biattarum***	**6**	6 (100%)									
**Intestinal protozoan parasitic infection in SOT (n 350)**											
***Cryptosporidium* spp.**	**210**	177 (84.3%)	11 (5.2%)	1 (0.5%)	1 (0.5%)		7 (3.3%)				13 (6.2%)
***Blastocystis* spp.**	**32**	31 (96.9%)									1 (3.1%)
***Giardia* spp.**	**18**	8 (44.4%)								1 (5.6%)	9 (50%)
***Entamoeba histolytica***	**2**		1 (50%)								1 (50%)
***Entamoebae* spp.**	**10**	10 (100%)									
***Chilomastix mesnili***	**1**	1 (100%)									
***Microsporidia* spp.**	**77**	50 (64.9%)	10 (13%)	1 (1.3%)	1 (1.3%)				1 (1.3%)	3 (3.9%)	11 (14.3%)
**Intestinal helminthic parasitic infection in SOT (n 78)**											
***Strongyioides stercoralis***	**72**	38 (52.8%)	6 (8.3%)	5 (6.9%)	2 (2.8%)	1 (1.4%)	2 (2.8%)			5 (6.9%)	13 (18.1%)
***Taenia soliun***	**2**		2 (100%)								
***Trichuris trichiura***	**1**	1 (100%)									
***Ascaris lumbricoides***	**2**	2 (100%)									
***Dipylidium caninum***	**1**	1 (100%)									
**Non-intestinal helminthic parasitic infection in SOT (n 14)**											
***Schistosoma* spp.**	**6**		6 (100%)								
***Echinococcus granulosus***	**2**	2 (100%)									
***Echinococcus multilocularis***	**6**	1 (16.7%)		5 (83.3%)							

**Table 2:** Post-SOT parasitosis grouped by donor and recipient characteristics.

Infectious Agent	Post-SOT Parasitosis (Total Number of Reported Cases)	Country of Origin or Nationality and/or Reported Risk Factors (e.g., Immigration/Travel History), n (%)											
		Donor						Recipent					
		North America	South/Central America	Africa	Asia	EU/EAA	n.a.	North America	South/Central America	Africa	Asia	EU/EAA	n.a.
**Non-intestinal protozoan parasitic infection in SOT (n 465)**													
***Toxoplasma gondii***	**162**					1 (0.6%)	161 (99.4%)				1 (0.6%)		161 (99.4%)
***Leishmania* spp.**	**151**						151 (100%)						151 (100%)
***Trypanosoma cruzi***	**88**	5 (5.7%)	17 (19.3%)				66 (75%)	3 (3.4%)	5 (5.7%)			1 (1.1%)	79 (89.8%)
***Plasmodium* spp.**	**27**			5 (18.5)	3 (11.1%)		19 (70.4%)				2 (7.4%)		25 (92.6%)
***Babesia***	**5**	3 (60%)					2 (40%)	3 (60%)					2 (40%)
***Acanthamoeba* spp.**	**17**						17 (100%)	2 (11.8%)					15 (88.2%)
***Balamuthia mandrillaris***	**9**	4 (44.4%)					5 (55.6%)						9 (100%)
***Lophomonas biattarum***	**6**						6 (100%)				2 (33.3%)		4 (66.7%)
**Intestinal protozoan parasitic infection in SOT (n 350)**													
***Cryptosporidium* spp.**	**210**						210 (100%)						210 (100%)
***Blastocystis* spp.**	**32**						32 (100%)						32 (100%)
***Giardia* spp.**	**18**						18 (100%)						18 (100%)
***Entamoeba histolytica***	**2**						2 (100%)						2 (100%)
***Entamoebae* spp.**	**10**						10 (100%)						10 (100%)
***Chilomastix mesnili***	**1**						1 (100%)						1 (100%)
***Microsporidia* spp.**	**77**					1 (1.3%)	76 (98.7%)						77 (100%)
**Intestinal helminthic parasitic infection in SOT (n 78)**													
***Strongyioides stercoralis***	**72**	3 (4.2%)	28 (38.9%)		9 (12.5%)		32 (44.4%)	7 (9.7%)	15 (20.8%)	1 (1.4%)	9 (12.5%)	3 (4.2%)	37 (51.4%)
***Taenia soliun***	**2**						2 (100%)		1 (50%)				
***Trichuris trichiura***	**1**						1 (100%)						1 (100%)
***Ascaris lumbricoides***	**2**						2 (100%)						2 (100%)
***Dipylidium caninum***	**1**						1 (100%)						1 (100%)
**Non-intestinal helminthic parasitic infection in SOT (n 14)**													
***Schistosoma* spp.**	**6**						6 (100%)			2 (33.3%)			4 (66.7%)
***Echinococcus granulosus***	**2**						2 (100%)				1 (50%)		1 (50%)
***Echinococcus multilocularis***	**6**						6 (100%)						6 (100%)

**Table 3:** Post-SOT parasitosis grouped by parasitic acquisition way.

Infectious Agent	Post-SOT Parasitosis (Total Number of Reported Cases)	Acquisition Mode, n (%)			Reactivation, n (%)	Not Available, n (%)
		Natural	Graft	Transfusion		
**Non-intestinal protozoan parasitic infection in SOT (n 465)**						
***Toxoplasma gondii***	**162**	16 (9.9%)	51 (31.5%)		13 (8%)	82 (50.6%)
***Leishmania* spp.**	**151**	1 (0.7%)			5 (3.3%)	145 (96%)
***Trypanosoma cruzi***	**88**	2 (2.3%)	29 (32.9%)		49 (55.7%)	8 (9.1%)
***Plasmodium* spp.**	**27**	1 (3.7%)	11 (40.7%)	1 (3.7%)		14 (51.8%)
***Babesia***	**5**		2 (40%)	2 (40%)		1 (20%)
***Acanthamoeba* spp.**	**17**					17 (100%)
***Balamuthia mandrillaris***	**9**		9 (100%)			
***Lophomonas biattarum***	**6**	2 (33.3%)				4 (66.7%)
**Intestinal protozoan parasitic infection in SOT (n 350)**						
***Cryptosporidium* spp.**	**210**				7 (3.3%)	203 (96.7%)
***Blastocystis* spp.**	**32**					32 (100%)
***Giardia* spp.**	**18**		1 (5.6%)			17 (94.4%)
***Entamoeba histolytica***	**2**					2 (100%)
***Entamoebae* spp.**	**10**					10 (100%)
***Chilomastix mesnili***	**1**					1 (100%)
***Microsporidia* spp.**	**77**					77 (100%)
**Intestinal helminthic parasitic infection in SOT (n 78)**						
***Strongyioides stercoralis***	**72**		37 (51.4%)		2 (2.8%)	33 (45.8%)
***Taenia soliun***	**2**				1 (50%)	1 (50%)
***Trichuris trichiura***	**1**					1 (100%)
***Ascaris lumbricoides***	**2**					2 (100%)
***Dipylidium caninum***	**1**					1 (100%)
**Non-intestinal helminthic parasitic infection in SOT (n 14)**						
***Schistosoma* spp.**	**6**		4 (66.7%)		2 (33.3%)	
***Echinococcus granulosus***	**2**				1 (50%)	1 (50%)
***Echinococcus multilocularis***	**6**			5 (83.3%)		1 (16.7%)

**Table 4:** Post-SOT parasitosis grouped by parasitic disease onset from transplantation.

Infectious Agent	Post-SOT (Total Number of Reported Cases)	Parasitic Disease Onset, n (%)			
		0–29 d PT	30–100 d PT	>100 d PT	n.a.
**Non-intestinal protozoan parasitic infection in SOT (n 465)**					
***Toxoplasma gondii***	**162**	7 (4.3%)	60 (37.1%)	14 (8.6%)	81 (50%)
***Leishimania* spp.**	**151**			23 (15.2%)	128 (84.8%)
***Trypanosoma cruzi***	**88**	1 (1.1%)	23 (26.1%)	6 (6.8%)	58 (66%)
***Plasmodium* spp.**	**27**	3 (11.2%)	13 (48.1%)		11 (40.7%)
***Babesia***	**5**		3 (60%)		2 (40%)
***Acanthamoeba* spp.**	**17**			3 (17.6%)	14 (82.4%)
***Balamuthia mandrillaris***	**9**	3 (33.3%)			6 (66.7%)
***Lophomonas biattarum***	**6**		4 (66.7%)	2 (33.3%)	
**Intestinal protozoan parasitic infection in SOT (n 350)**					
***Cryptosporidium* spp.**	**210**	1 (0.5%)		64 (30.5%)	145 (69%)
***Blastocystis* spp.**	**32**				32 (100%)
***Giardia* spp.**	**18**				18 (100%)
***Entamoeba histolytica***	**2**				2 (100%)
***Entamoebae* spp.**	**10**				10 (100%)
***Chilomastix mesnili***	**1**				1 (100%)
***Microsporidia* spp.**	**77**		4	24	49
**Intestinal helminthic parasitic infection in SOT (n 78)**					
***Strongyioides stercoralis***	**72**	4 (5.5%)	20 (27.8%)	9 (12.5%)	39 (54.2%)
***Taenia soliun***	**2**		1 (50%)	1 (50%)	
***Trichuris trichiura***	**1**				1 (100%)
***Ascaris lumbricoides***	**2**				2 (100%)
***Dipylidium caninum***	**1**			1 (100%)	
**Non-intestinal helminthic parasitic infection in SOT (n 14)**					
***Schistosoma* spp.**	**6**		1 (16.7%)		5 (83.3%)
***Echinococcus granulosus***	**2**			2 (100%)	
***Echinococcus multilocularis***	**6**			5 (83.3%)	1 (16.7%)

**Table 5:** Post-SOT parasitosis grouped by laboratory diagnostic methods.

Infectious Agent	Post-SOT Parasitosis (Total Number of Reported Cases)	Laboratories Diagnosis in Transplant Recipient, n (%)					
		Direct			Indirect	Both (Direct/Indirect)	n.a.
		Ante-Mortem	Post-Mortem	Both (ante/post)			
**Non-intestinal protozoan parasitic infection in SOT (n 465)**							
***Toxoplasma gondii***	**162**	11 (6.8%)	2 (1.2%)		18 (11.1%)	5 (3.1%)	126 (77.8%)
***Leishmania* spp.**	**151**	32 (21.2%)	1 (0.7%)		3 (1.9%)	48 (31.8%)	67 (44.4%)
***Trypanosoma cruzi***	**88**	24 (27.3%)	1 (1.1%)		5 (5.7%)	12 (13.6%)	46 (52.3%)
***Plasmodium* spp.**	**27**	22 (81.5%)			1 (3.7%)	1 (3.7%)	3 (11.1%)
***Babesia***	**5**					3 (60%)	2 (40%)
***Acanthamoeba* spp.**	**17**	2 (11.8%)	2 (11.8%)				13 (76.4%)
***Balamuthia mandrillaris***	**9**	1 (11.1%)	2 (22.2%)	2 (22.2%)		4 (44.5%)	
***Lophomonas biattarum***	**6**	6 (100%)					
**Intestinal protozoan parasitic infection in SOT (n 350)**							
***Cryptosporidium* spp.**	**210**	143 (68.1%)			2 (0.9%)	17 (8.1%)	48 (22.9%)
***Blastocystis* spp.**	**32**	29 (90.6%)					3 (9.4%)
***Giardia* spp.**	**18**						18 (100%)
***Entamoeba histolytica***	**2**						2 (100%)
***Entamoebae* spp.**	**10**						10 (100%)
***Chilomastix mesnili***	**1**						1 (100%)
***Microsporidia* spp.**	**77**	70 (90.9%)	2 (2.6%)			4 (5.2%)	1 (1.3%)
**Intestinal helminthic parasitic infection in SOT (n 78)**							
***Strongyioides stercoralis***	**72**	21 (29.1%)	1 (1.4%)	1 (1.4%)	1 (1.4%)		48 (66.7%)
***Taenia soliun***	**2**	1 (50%)			1 (50%)		
***Trichuris trichiura***	**1**	1 (100%)					
***Ascaris lumbricoides***	**2**	2 (100%)					
***Dipylidium caninum***	**1**	1 (100%)					
**Non-intestinal helminthic parasitic infection in SOT (n 14)**							
***Schistosoma* spp.**	**6**	6 (100%)					
***Echinococcus granulosus***	**2**	1 (50%)					1 (50%)
***Echinococcus multilocularis***	**6**				1 (16.7%)	5 (83.3%)	

**Table 6:** Post-SOT parasitosis grouped by parasitic disease treatment and patient outcome *.

Infectious Agent	Post-SOT Parasitosis (Total Number of Reported Cases)	Parasitic Disease Treatment, n (%)				Outcome, m (%)			
		Standard Treatment	Alternative Regimen	Not Done	n.a	Recovery	Death	Replace	n. a
**Non-Intestinal protozoan parasitic infections in SOT (n 465)**									
***Toxoplasma gondii***	**162**	76 (46.9%)			86 (53.1%)	50 (30.9%)	22 (13.6%)	1 (o.6%)	89 (54.9%)
***Leishmania* spp.**	**151**	60 (39.7%)			91 (60.3%)	34 (22.5%)	8 (8.3%)	58 (38.4%)	51 (33.8%)
***Trypanosoma cruzi***	**88**	33 (37.5%)		4 (4.5%)	51 (58%)	16 (18.2%)	20 (22.7%)	2 (2.3%)	50 (56.8%)
***Plasmodium* spp.**	**27**	17 (63%)			10 (37%)	20 (74.1%)	1 (3.7%)		6 (22.2%)
***Babesia***	**5**	4 (80%)			1 (20%)	3 (60%)			2 (40%)
***Acanthamoeba* spp.**	**17**				17 (100%)	5 (29.4%)	11 (67.4%)		1 (5.9%)
***Balamuthia mandrillaris***	**9**	6 (66.7%)		1 (11.1%)	2 (22.2%)	5 (55.6%)	4 (44.4%)		
***Lophomonas biattarum***	**6**	2 (33.3%)			4 (66.7%)	2 (33.3%)			4 (66.7%)
**Intestinal protozoan parasitic infections in SOT (n 350)**									
***Cryptosporidium* spp.**	**210**	185 (88.1%)	1 (0.5%)		24 (11.4%)	176 (83.8%)	3 (1.4%)	7 (3.4%)	24 (11.4%)
***Blastocystis* spp.**	**32**	2 (6.2%)			30 (93.8%)				32 (100 %)
***Giardia* spp.**	**18**				18 (100%)	1 (5.6%)			17 (94.4%)
***Entamoeba histolytica***	**2**	1 (50%)			1 (50%)	1 (50%)			1 (50%)
***Entamoebae* spp.**	**10**				10 (100%)				10 (100%)
***Chilomastix mesnili***	**1**				1 (100%)				1 (100%)
***Microsporidia* spp.**	**77**	52 (67.5%)	1 (1.3%)	4 (5.2%)	20 (26%)	28 (36.4%)	4 (5.2%)	2 (2.6%)	43 (55.8%)
**Intestinal protozoan parasitic infections in SOT (n 78)**									
***Strongyioides stercoralis***	**72**	34 (45.8%)			39 (54.2%)	37 (51.4%)	23 (31.9%)	2 (2.8%)	10 (13.9%)
***Taenia soliun***	**2**	1 (50%)	1 (50%)			2 (100%)			
***Trichuris trichiura***	**1**	1 (100%)				1 (100%)			
***Ascaris lumbricoides***	**2**				2 (100%)				2 (100%)
***Dipylidium caninum***	**1**	1 (100%)				1 (100%)			
**Non-Intestinal protozoan parasitic infections in SOT (n 14)**									
***Schistosoma* spp.**	**6**	2 (33.3%)		4 (66.7%)		4 (66.7%)	2 (33.3%)		
***Echinococcus granulosus***	**2**	2 (100%)				2 (100%)			
***Echinococcus multilocularis***	**6**	2 (88.3%)			1 (16.7%)	3 (50%)	2 (33.3%)		1 (16.7%)

* Out of all reported cases with specified standard or alternative treatment regimens, around 76% had favorable outcome with recovery, about 12% had negative outcome with death, whereas about 6% experienced recurrence, with the remaining having unspecified outcome.

## Abbreviations

BAL	bronchoalveolar lavage
CNI	calcineurin inhibitors
CNS	central nervous system
CsA	Cyclosporine
CSF	cerebral spinal fluid
CMV	Cytomegalovirus
D+/D−	*Toxoplasma*-seropositive/seronegative donor
HSCT	allogeneic haematopoietic stem cell transplant
mTORI	mammalian target of rapamycin inhibitors
NCC	Neurocysticercosis
PCR	polymerase chain reaction
PTLD	post-transplant lymphoproliferative disorder
R+/R−	*Toxoplasma*-seropositive/seronegative recipient
SOLs	intracranial space-occupying lesions
SOT	solid organ transplantation
TAC	Tacrolimus
TB	Tuberculosis
TMP-SMZ	trimethoprim-sulfamethoxazole
WB	western blot
